# Multisensory stimulation improves functional recovery and resting-state functional connectivity in the mouse brain after stroke

**DOI:** 10.1016/j.nicl.2017.11.022

**Published:** 2017-12-02

**Authors:** Jakob Hakon, Miriana Jlenia Quattromani, Carin Sjölund, Gregor Tomasevic, Leeanne Carey, Jin-Moo Lee, Karsten Ruscher, Tadeusz Wieloch, Adam Q. Bauer

**Affiliations:** a Laboratory for Experimental Brain Research, Division of Neurosurgery, Department of Clinical Sciences, Lund University, BMC A13, 22184 Lund, Sweden; b Department of Neurosurgery, University Hospital of Lund, Lund, Sweden; c School of Allied Health, La Trobe University, Melbourne, Vic., Australia; d Neurorehabilitation and Recovery Laboratory, Florey Institute of Neuroscience and Mental Health, Melbourne, Vic., Australia; e Department of Radiology, Washington University, Saint Louis, MO 63110, USA; f Department of Neurology, Washington University, Saint Louis, MO 63110, USA; g Department of Biomedical Engineering, Washington University, Saint Louis, MO 63110, USA

**Keywords:** RS-FC, resting-state functional connectivity, fcOIS, functional connectivity optical intrinsic signal imaging, GSR, global signal regression, MSR, multiple signal regression, ROI, region of interest, ND*i*, intrahemispheric node degree, ND*c*, interhemispheric (contralateral) node degree, STD, standard environment, EE, enriched environment, PV, parvalbumin, M1, primary motor cortex, M2, secondary motor cortex, M2p, posterior secondary motor cortex, SFL, somatosensory forelimb cortex, PP, posterior parietal cortex, RS, retrosplenial cortex, VIS, visual cortex, Resting-state functional connectivity, Optical imaging, Stroke, Recovery, Enriched environment, Parvalbumin

## Abstract

Stroke causes direct structural damage to local brain networks and indirect functional damage to distant brain regions. Neuroplasticity after stroke involves molecular changes within perilesional tissue that can be influenced by regions functionally connected to the site of injury. Spontaneous functional recovery can be enhanced by rehabilitative strategies, which provides experience-driven cell signaling in the brain that enhances plasticity. Functional neuroimaging in humans and rodents has shown that spontaneous recovery of sensorimotor function after stroke is associated with changes in resting-state functional connectivity (RS-FC) within and across brain networks. At the molecular level, GABAergic inhibitory interneurons can modulate brain plasticity in peri-infarct and remote brain regions. Among this cell-type, a decrease in parvalbumin (PV)-immunoreactivity has been associated with improved behavioral outcome. Subjecting rodents to multisensory stimulation through exposure to an enriched environment (EE) enhances brain plasticity and recovery of function after stroke. Yet, how multisensory stimulation relates to RS-FC has not been determined. In this study, we investigated the effect of EE on recovery of RS-FC and behavior in mice after stroke, and if EE-related changes in RS-FC were associated with levels of PV-expressing neurons. Photothrombotic stroke was induced in the sensorimotor cortex. Beginning 2 days after stroke, mice were housed in either standard environment (STD) or EE for 12 days. Housing in EE significantly improved lost tactile-proprioceptive function compared to mice housed in STD environment. RS-FC in the mouse was measured by optical intrinsic signal imaging 14 days after stroke or sham surgery. Stroke induced a marked reduction in RS-FC within several perilesional and remote brain regions. EE partially restored interhemispheric homotopic RS-FC between spared motor regions, particularly posterior secondary motor. Compared to mice housed in STD cages, EE exposure lead to increased RS-FC between posterior secondary motor regions and contralesional posterior parietal and retrosplenial regions. The increased regional RS-FC observed in EE mice after stroke was significantly correlated with decreased PV-immunoreactivity in the contralesional posterior motor region. In conclusion, experimental stroke and subsequent housing in EE induces dynamic changes in RS-FC in the mouse brain. Multisensory stimulation associated with EE enhances RS-FC among distinct brain regions relevant for recovery of sensorimotor function and controlled movements that may involve PV/GABA interneurons. Our results indicate that targeting neural circuitry involving spared motor regions across hemispheres by neuromodulation and multimodal sensory stimulation could improve rehabilitation after stroke.

## Highlights

• Disconnection within and across functional networks in the mouse brain after stroke is reduced following multisensory stimulation by enriched environment (EE).
• Multisensory stimulation by EE enhances functional connectivity among distinct brain regions relevant for recovery of controlled movement and tactile-proprioception function.
• Increased regional RS-FC observed in stimulated mice after stroke was significantly correlated with decreased regional parvalbumin-immunoreactive cell count.
• Cortical regions related to motor control are possible targets for neuromodulation after stroke.

## 1 Introduction

Stroke is a leading cause of death and long term adult disability (Mozaffarian et al., 2016). Following stroke, direct tissue damage and disconnection of remote brain areas causes functional disruption that can span multiple domains (Carter et al., 2010, He et al., 2007, Silasi and Murphy, 2014). Neuroplasticity after stroke involves molecular changes within perilesional and tissue remote from the lesion that can be influenced by distant regions spared from injury (Cramer, 2008). Understanding and influencing the processes of functional neuroplasticity are therefore crucial in providing more effective therapies.

Recovery of sensorimotor function can be enhanced by rehabilitative training of the affected modality (Carey et al., 2011, Wolf et al., 2010). Other complex multimodal training paradigms have also shown efficacy in accelerating rehabilitation outcomes (Bunketorp-Käll et al., 2017). Non-invasive brain stimulation strategies, such as transcranial magnetic stimulation and direct current stimulation, provide support for rehabilitation interventions (Hatem et al., 2016), and mimic aspects of peripheral stimulation that might trigger endogenous plasticity mechanisms of the brain (Carmichael, 2012, Wieloch and Nikolich, 2006). In animal models of stroke, multisensory stimulation can be applied through enriched environments (EE), i.e. cages of 5–10 animals containing multilevel platforms, tubes, chains and toys (Johansson and Ohlsson, 1996). The complex task- and experience-driven stimulation of brain plasticity following exposure to EE affects spine density and size, release of growth factors, and changes in cell signaling in the brain considered to improve sensory, motor, and cognitive function (Nithianantharajah and Hannan, 2006).

At the systems level, functional magnetic resonance imaging (fMRI) has revealed that behavioral recovery is associated with changes in patterns of resting-state functional connectivity (RS-FC) within and across resting-state networks (Carey et al., 2013). FMRI studies in humans shortly after ischemic stroke demonstrated that disruption of interhemispheric homotopic RS-FC predicted poor motor, somatosensory, and attentional recovery (Bannister et al., 2015, Carter et al., 2010). In a mouse model of stroke, functional connectivity optical intrinsic signal imaging (fcOIS) revealed that disruption of homotopic RS-FC correlates with infarct size and acute behavioral deficits (Bauer et al., 2014). A similar fMRI study in rats after stroke revealed that restoration of homotopic RS-FC correlated with spontaneous improvement in chronic sensorimotor function (van Meer et al., 2010).

At the molecular level, multiple mechanisms contribute dynamically to the post stroke recovery process over multiple spatiotemporal scales (Wieloch and Nikolich, 2006). For example, GABAergic inhibitory interneurons modulate brain plasticity in the perilesional and remote brain regions by affecting the balance between excitation and inhibition in the recovery phase after stroke (Clarkson et al., 2010, Witte, 2000). Among the population of GABAergic interneurons, parvalbumin (PV)-immunoreactive cells are of particular importance. These cells are considered gate keepers of excitability of the cortical microcolumns and regulators of cortical gamma oscillations important for cognition and brain plasticity (Freund, 2003, Hensch, 2004). Enriched housing decrease hippocampal PV immunoreactivity in PV/GABA cells, indicating that these cells adopted an early developmental phenotype seen during critical periods of brain development (Donato et al., 2013). After experimental stroke, PV levels are modulated (Alia et al., 2016, Inácio et al., 2011) and a decrease in the levels of PV-immunoreactivity has been associated with improved functional recovery (Zeiler et al., 2013). A large fraction of cortical PV inhibitory neurons have been recently demonstrated to exhibit long-range transcallosal projections interconnecting homotopic cortical regions, supporting a direct role of PV^+^ cells on interhemispheric inhibition (Rock et al., 2017), which might also influence recovery after stroke.

Despite the number of studies describing the therapeutic effects of EE on behavioral recovery, information concerning how EE influences systems- and molecular-level changes in the brain remains lacking. We hypothesized that multisensory stimulation of the brain by EE housing after stroke leads to improved functional recovery and RS-FC after stroke. To test this hypothesis, we employed fcOIS to study changes within various functional cortical networks in mice after photothrombotic stroke involving the left sensorimotor cortex. Two days after stroke, mice were moved to EE housing or standard (STD) cages. FcOIS was performed 14 days after stroke or sham surgery, and behavioral outcome was evaluated at 2 and 14 days of recovery. Brains were harvested on day 14 after the final imaging session. To evaluate molecular changes in remodeled cortex, populations of PV-immunoreactive neurons were examined using confocal microscopy.

## 2 Materials and methods

### 2.1 Ethical statement

All animal procedures were approved by the Malmö-Lund ethical committee (permit number: M 50-15) and reported according to the ARRIVE guidelines. The mice were housed in STD laboratory cages (2 mice per cage) before stroke or sham surgery, and in STD or EE after stroke (see “Housing conditions” section below for details). The mice were kept in a reverse light-cycle with free access to food and water. Image acquisition and behavioral analysis were performed during the awake periods.

### 2.2 Surgical procedures

#### 2.2.1 Mounting of glass window

Fifty-two C57bl mice aged 8–10 weeks were included in the study. One week prior to stroke mice were anesthetized with 2% isoflurane and mounted in a stereotactic frame. A midline skin incision was made to expose the skull. A round coverslip glass plate 13 mm in diameter and 0.15 mm in thickness was glued on top of the exposed skull with Super-Bond (L-type Clear, Sun Medical), and the skin was glued at the edges of the glass window to close the incision.

#### 2.2.2 Induction of photothrombosis

For induction of photothrombotic stroke, mice were anesthetized using isoflurane (2% in O_2_) and positioned in a stereotaxic frame. Body temperature was maintained with a heating pad set to a target temperature of 37 **°**C. A bolus of Rose Bengal (0.1 mg/kg, Sigma) was injected in the peritoneal cavity. Ten minutes after the injection, a 4 × 2 mm^2^ rectangular area (localized 2.5 to − 1.5 mm from bregma, beginning 0.8 mm to the left of the midline) was illuminated for 20 min with 3100 K light (Schott, KL 1500). Sham surgeries were performed similarly without illumination following injection of Rose Bengal. All mice recovered on a heating pad after the stroke and sham procedures.

### 2.3 Housing conditions

Two days after stroke and sham surgeries, all mice were sorted in pairs into either STD or EE cages where they were housed for 12 days. A researcher who was not involved in the stroke procedure or behavior assessments performed the sorting. Fifteen mice with stroke and 11 sham mice were housed in STD cages; 15 mice with stroke and 11 mice with sham surgery were housed in EE cages as described previously (Nygren and Wieloch, 2005, Quattromani et al., 2014). Each EE cage measured 40 cm × 40 cm × 35 cm, and contained 5–6 mice. Sham and stroke mice were housed together in EE cages. The EE cages were equipped with tubes, chains, ladders, toys and platforms at different levels. Objects were rearranged twice a week.

### 2.4 The paw-placement test

At 2 and 14 days of recovery, sensorimotor function of each paw was evaluated by the paw-placement test (De Ryck et al., 1992, Madinier et al., 2014). An observer blinded to the housing condition performed the paw-placement test. All mice were placed on a 6 mm thick platform and held in a horizontal grip allowing them to freely move all four paws. The head was angled 45 degrees upward so the mice would not have visual guidance of the platform or paw position, nor any contact between the whiskers and the platform. Mice were then moved laterally towards the edge of the platform until the two paws, towards the edge, lost contact with the surface. This protocol ensured that the tactile-proprioception demand of paw-placement was present (not compensated for) within this sensorimotor task. If a mouse responded by quickly moving the paw to the surface, it was assigned a score of 1. Incomplete placement of the paw on the platform, or if the paw supinated and moved inwards towards the platform edge, were assigned a score of 0.5. A score of 0 was given for a persistent free-hanging limb.

### 2.5 Optical intrinsic signal imaging

#### 2.5.1 Imaging system

The fcOIS system is based on a setup previously described (Bauer et al., 2014, White et al., 2011). Optical intrinsic signals were acquired during sequential elimination provided by four polarized light emitting diodes (LEDs, Thorlabs, 470 nm: M470L3-C1, 590 nm: M590L3-C1, 617 nm: M617L3-C1, and a 625 nm: M625L3-C1) placed approximately 20 cm above the mouse's head. Diffuse reflected light from the mouse head was detected by a cooled, frame-transfer EMCCD camera (iXon 897 Ultra, Andor Technologies). A crossed linear polarizer was placed in front of the camera lens to reject specular reflection from the LEDs. The LEDs and camera were time-synchronized and controlled via a National Instruments data Acquisition card (NI PCI 6733) and computer (Dell Workstation) using custom-written software (MATLAB, Mathworks) at a frame rate of 120 Hz (30 Hz/LED).

#### 2.5.2 Image acquisition

For each mouse, images were acquired 14 days after stroke and sham surgery. Anesthesia was initiated in a Plexiglas chamber with 4% isoflurane in O_2_:N_2_ (40:60). After induction, isoflurane was adjusted to 2% and mice were positioned in a stereotactic frame with a heating pad connected to a two-channel temperature controller set to 37 **°**C (Cell Micro Controls). Eyes were covered with eye gel and black tape. A 23G needle was inserted subcutaneously in the interscapular region, and connected to an infusion pump with a polyethylene tube. Approximately 10 min after anesthesia induction a subcutaneous bolus of 0.3 mg/kg Medetomidine (Domitor, Orion Pharma) was injected (Adamczak et al., 2010, Nasrallah et al., 2014). Isoflurane was gradually discontinued during the next 5–6 min and the breathing air mixture was changed to O_2_:N_2_ (30:70). Mice were then mounted underneath the camera. Ten minutes after bolus injection, constant infusion of Medetomidine (0.6 mg/kg/h) was initiated. Mice were allowed to stabilize for 15 min before imaging. Nine, 5-minute data sets were collected in each mouse.

#### 2.5.3 Image processing

A brain mask was delineated manually using a white-light image of the cortex for each mouse (Bauer et al., 2014). Only data within the brain mask was processed further. The midline junction between the olfactory bulb and cerebrum and lambda were defined for each mouse and all imaging data were affine-transformed into a common mouse atlas space (Paxinos and Franklin, 2001) as previously described (Bauer et al., 2014). Quality control was performed for each 5 min data run. Empirically, we have observed that movement associated with animals improperly secured to the mouse stage or waking from anesthesia produces relatively large fluctuations in light level compared to underlying brain activity. We therefore excluded any session with > 1% temporal variation in whole-brain, mean light level intensity for each wavelength. Spectroscopic analysis was then performed at each pixel to convert temporal changes in reflected light intensity into changes in oxy- and deoxy-hemoglobin as described previously (Bauer et al., 2014, White et al., 2011). Spontaneous hemodynamic brain activity was collected at 30 Hz to avoid aliasing of physiologic signals (e.g. respiration, pulse) occurring between 3 and 10 Hz. Hemodynamic activity associated with intrinsic brain organization (i.e. resting-state network fluctuations) generally occurs over frequencies < 0.1 Hz. Data were filtered (0.009 Hz–0.08 Hz) according to rodent (Bauer et al., 2014, van Meer et al., 2012) and human fMRI algorithms (Fox et al., 2005), and down-sampled from 30 Hz to 1 Hz to reduce file size. For sham day 14 imaging, global signal regression (GSR) was performed prior to RS-FC analysis to minimize shared variance over the cortex. In animals subjected to stroke, we used a multiple signal regression (MSR) approach to minimize the effects of temporal lag on measures of RS-FC (Bauer et al., 2014). For MSR, two regressors were calculated: one from averaging all time series within the infarct, and another from averaging the time series from the non-infarcted tissue. These 2 signals were regressed simultaneously from all brain pixels. The spatial extent of the infarct within our field-of-view was determined via histology as described in Section 2.7 below.

#### 2.5.4 Functional connectivity measurements

##### 2.5.4.1 Seed-based functional connectivity

**Supplementary Fig. 1:**
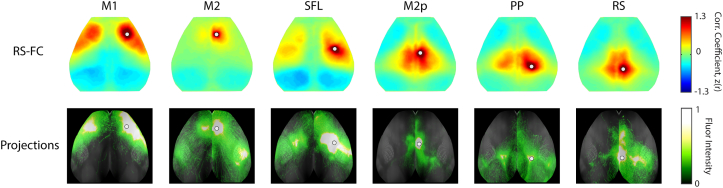
Seed-based functional connectivity in relation to cortical axonal projections. Seed-based functional connectivity compared to axonal projection connectivity from the Allen Mouse Brain Connectivity Atlas reveal strong correspondence between patterns of structural and functional connectivity in all major brain networks examined. Further, strong intra-network structural and functional connectivity exists between M2p, PP and RS areas, and between M1, M2 and SFL regions. Images are modified from experiments: 100,141,780 (M1 injection), 168,162,771 (M2 injection), 180,718,587 (SFL injection), 496,150,781 (M2p injection), 294,533,406 (PP association (anterior/medial) injection) and 100,148,142 (RS). Abbreviations: M1: Primary motor, M2 secondary motor, M2p: posterior M2, SFL: somatoszensory forelimb, PP: posterior parietal, RS: retrosplenial.

**Supplementary Fig. 2:**
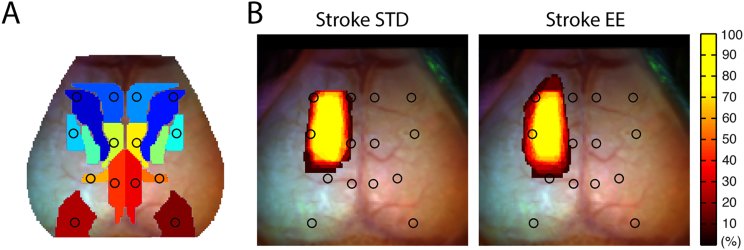
Incidencemaps and seed location. For both groups the perilesional primary motor seed is located in the 20%–50% lesion incidence range, with the center at 33%. The somatosensory hindlimb seed is centered in the 40–50% range for both groups. Secondary motor seeds are below the 10% range for both groups.

Fourteen regions of interest (seeds) with a diameter of 0.25 mm were placed in both hemispheres. We were primarily interested in the effects of multisensory stimulation on local and distant functional connectivity of regions known to be involved in sensorimotor processing but spared from direct injury. We therefore chose to examine regions of interest (ROIs) within perilesional tissue and cortical regions unaffected by focal damage but relevant for sensorimotor function. ROIs in unlesioned brain regions are positioned at approximately the center-of-mass of the functional region as defined by the Paxinos atlas. This location corresponds to where the peak functional response occurs following sensory stimulation (e.g. peripheral stimulation of the forepaw (Bauer et al., 2014)); a location expected to be accurate for other regions that are more difficult to activate with sensory paradigms (e.g. retrosplenial cortex) (Mohajerani et al., 2011, Lim et al., 2014). Secondary motor was subdivided into anterior and posterior regions, based on the axonal projection connectivity profile of anatomical tracers injected into these regions (Fig. S1). Positions for perilesional seeds were determined by overlapping the lesion incidence map with the Paxinos atlas. Cortical regions having 25% or more area spared from direct injury across all mice (> 50% incidence) were considered perilesional. Perilesional seed positions were placed in the center of the remaining region spared from direct injury. All seed positions relative to lesion incidence are reported in Fig. S2. From each seed region, time traces of oxygenated hemoglobin were correlated with time courses in all brain pixels, generating an RS-FC map for each seed. Individual mouse RS-FC maps were transformed to Fisher z-scores and averaged within each group. Seed-based RS-FC maps are displayed using a color scale where negative correlations are in blue hues and positive correlations in red hues. RS-FC matrices were constructed by compressing RS-FC maps to report RS-FC between each seed-seed pair. Maps of cortical projection connectivity were collected from the Allen Mouse Brain Connectivity Atlas (Allen Institute for Brain Science, 2014, Oh et al., 2014) (Fig. S1). Images are modified from experiments: 100141780 (primary motor injection), 168162771 (secondary motor injection), 180718587 (somatosensory forelimb injection), 496150781 (posterior secondary motor injection), 294533406 (posterior parietal association (anterior/medial) injection), 100148142 (retrosplenial) and 307137980 (primary visual). The strongest overlap between structural and functional connectivity patterns are observed between posterior secondary motor, posterior parietal and retrosplenial areas, and in networks including primary motor, secondary motor, and somatosensory forelimb regions. In addition, visual regions have strong anticorrelations towards primary motor, secondary motor and somatosensory forelimb. Changes in interregional RS-FC between groups were quantified for seeds within two areas (1: primary and secondary motor, somatosensory and visual; 2: posterior secondary motor, posterior parietal and retrosplenial), yielding a total of 42 comparisons.

##### 2.5.4.2 Homotopic functional connectivity

Interhemispheric RS-FC between homotopic brain regions was calculated by correlating the time trace of each pixel in the right hemisphere with the time trace of its contralateral homolog in the left hemisphere (i.e. mirrored brain pixels about midline). This procedure generated a brain-wide homotopic RS-FC map. Because homotopic RS-FC maps are mathematically symmetric (if A is correlated with B, then B is correlated with A), we chose to only visualize these maps in the ipsilesional (left) hemisphere. Values of Fisher z-transformed Pearson correlations for each mouse were averaged to generate group-level homotopic RS-FC maps. Difference maps were calculated between EE and STD conditions (EE minus STD) for both sham and stroke groups.

##### 2.5.4.3 Regional intra- and inter-hemispheric node degree

**Fig. 1:**
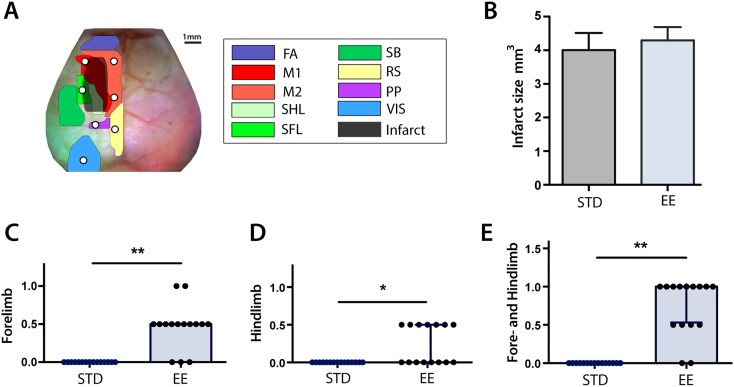
Imaging field-of-view, infarct size, and tests of tactile-proprioceptive function. (A) Dorsal view of the mouse brain; functional assignments within our field-of-view are color-coded according to Paxinos and Franklin (2001). White dots indicate seed location and size. The stroke lesion (black) indicates the 50% lesion incidence of all mice. (B) Infarct size 14 days after stroke (*n* = 15 mice in each housing condition; *p* = 0.66). Paw-placement scores of (C) contralesional forelimb, (D) contralesional hindlimb and (E) combined contralesional forelimb and hindlimb 14 days after stroke. Scores are shown as individual data points with group medians and error bars showing upper and lower quartiles (*p < 0.01, **p < 0.001; Mann-Whitney test). Abbreviations: FA: frontal association, M1: primary motor, M2: secondary motor, SHL: somatosensory hindlimb, SFL: somatosensory forelimb, SB: somatosensory barrel, RS: retrosplenial, PP: posterior parietal, VIS: visual, STD: standard environment, EE: enriched environment.

Correlating each pixel's time course with the time courses of all other pixels in the brain yields a matrix containing all of the RS-FC information within our field-of-view. Specifically, each row or column of this matrix represents a pixel's RS-FC map. The number of functional connections (degree) of each pixel (node) was determined by thresholding this matrix at z(r) ≥ 0.4, so that only strong positive RS-FC contributed to each pixel's measure of node degree (Buckner et al., 2009). Summing the correlation coefficients above this threshold over pixels in the same (ipsilateral) or opposite (contralateral) hemisphere relative to a candidate pixel produced a weighted measure of intra- or inter-hemispheric node degree for that pixel, respectively. This procedure was performed in each mouse over pixels within the shared brain mask. Group-level maps were created by averaging intra- and inter-hemispheric node degree maps across mice, and quantified within brain regions defined by the Paxinos atlas (Fig. 1).

### 2.6 Preparation of brain tissue

After the final imaging session (day 14), mice were anesthetized with isoflurane and transcardially perfused with saline followed by cold 4% paraformaldehyde (PFA). Brains were removed from the skull, immersed in 4% PFA overnight and then in 25% sucrose solution for 48 h. Brains were sectioned into 30-μm coronal free-floating sections with a sliding microtome and stored in antifreeze solution for further histochemical processing.

### 2.7 Immunohistochemestry and infarct evaluation

For each mouse, one free-floating section every 0.5 mm was collected for determination of infarct size. All sections were washed in PBS and quenched (3% H_2_O_2_ and 10% MeOH) for 12 min. After quenching, sections were blocked for 1 h in a blocking solution containing 5% normal donkey serum and 0.25% Triton X-100, and thereafter incubated in rabbit monoclonal anti-NeuN antibody 1:5000 (MABN140, Millipore) at 4 °C overnight. After washing, the sections were incubated with a secondary biotinylated donkey anti-rabbit antibody 1:400 (Jackson ImmunoResearch) for 1.5 h. Immunohistochemical visualization was performed using a Vectorstain ABC kit (Vectorlab) and 3,3-diaminobenzidine/H_2_O_2_ (DabSafe, Saveen Werner). Finally, NeuN-stained sections were mounted on a microscope glass, and imaged using a flatbed scanner. The area of intact NeuN-stained tissue was determined for each section using the ImageJ software. Infarcted area of grey matter for each section was determined by subtracting the area of intact tissue of the contralesional hemisphere from the ipsilesional side for each section. Infarct volume was calculated by integration (Swanson et al., 1990).

For quantification of NeuN positive cells, sections were photographed at 10 × magnification using a brightfield microscope (Olympus BX60, Solna, Sweden). The percentage of NeuN positive cells in the posterior motor regions were measured by thresholding for dark objects indicative of immunoreactive cells (ImageJ, iterative intermeans algorithm). The area NeuN specific immunoreactivity within the region of interest was presented as percentage of the total area.

**Supplementary Fig. 3:**
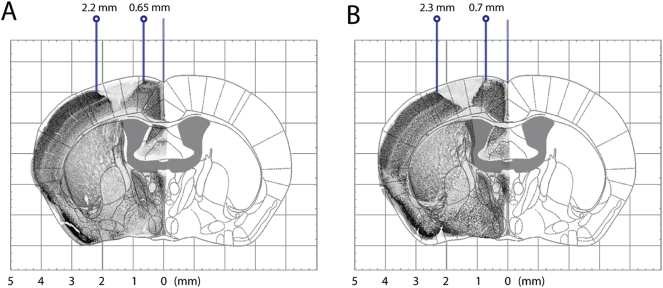
Representative histological images of NeuN stained brain slices (− 0.2 mm from bregma) of the (A) stroke STD and (B) stroke EE group. The slices are overlaid on an atlas grid to illustrate how the coordinates for lateral and medial lesion borders were determined. The atlas was adapted from the Paxinos atlas (Paxinos and Franklin, 2001).

For lesion masking, images of all NeuN-stained slices were transformed and overlaid on the correspondent atlas template in Adobe Photoshop. Medial and lateral coordinates of the infarcts were noted and used to create a 2D lesion masks for each stroke mouse (representative images shown in Fig. S3).

### 2.8 Immunofluorescence

Free-floating slices at bregma level − 0.2 mm were washed in PBS and incubated in blocking solution (5% normal donkey serum and 0.25% Triton X-100) for 1 h at RT. Sections were incubated over night with the primary antibody goat anti-PV (1:2500, PVG-214 Swant, Marly Switzerland) in blocking solution. Following washings, the sections were incubated with Cy3-conjugated donkey anti-goat (1:400, Jackson ImmunoResearch, Suffolk UK) for 1.5 h at RT. Fluorescent dyes were imaged with a confocal laser-scanning microscope (Zeiss LSM 510, Germany). Cell counting was performed within the region of interest determined by projecting the anatomical atlas over the scanned images.

### 2.9 Statistical analysis

Statistical analyses of RS-FC measures were performed using MATLAB. Prior to any statistical testing, Pearson *r* values were transformed to Fisher *z*-scores. For biochemical data, statistical analysis was determined using Prism GraphPad 7. When comparing two groups, a two-tailed unpaired *t*-test was used, except for nonparametric paw-placement data for which a Mann-Whitney *U* test was used. For analyzing differences between groups in the seed based matrices, a *t*-test was followed by FDR correction for multiple comparisons. When comparing more than two groups, a one-way ANOVA was applied, and followed by a FDR correction for multiple comparisons. Statistical comparisons of node degree between groups in several regions was done using a two-way ANOVA with group serving as one variable and brain region as a repeated measure, followed by a FDR post hoc test. Differences between groups were considered statistically significant if *p* < 0.05 following correction for multiple comparisons. Data are presented as mean ± SEM.

## 3 Results

### 3.1 Infarct size and paw-placement ability

Photothrombotic stroke caused an infarct including left primary motor and secondary motor areas and forelimb and hindlimb somatosensory cortical areas (Fig. 1A). There was no difference in infarct size between mice housed in STD (4.0 ± 0.5 mm^3^, *n* = 15) and EE (4.3 ± 0.4 mm^3^, *n* = 15, *p* = 0.66, Fig. 1B) 14 days after stroke. Thus, placing animals into EE starting 2 days after stroke did not affect the size of the infarct twelve days later, consistent with previous work in rats and mice (Johansson and Ohlsson, 1996, Madinier et al., 2014, Quattromani et al., 2014).

Forelimb and hindlimb tactile-proprioceptive function assessed by the paw-placement test was markedly affected by stroke (Fig. 1C–E). Sham animals did not show any deficits after sham surgery (data not shown). All mice subjected to stroke showed full deficit in paw placement function 2 days after stoke (score = 0 of both right-sided limbs). Housing mice in EE from day 2 to day 14 after stroke significantly improved paw-placement ability of affected right forelimb (Fig. 1C) and hindlimb (Fig. 1D) compared to mice in STD cages. Eighty-seven percent of the mice in EE showed some degree of recovery of right-sided extremities in contrast to 0% in the STD group (Fig. 1E). Eighty-percent of the mice housed in EE recovered in forelimb function, whereas only 47% recovered in hindlimb function. Two mice housed in EE fully recovered forelimb paw-placement function. None of the mice achieved full recovery of hindlimb function. Together, these results showed that EE improved paw placement function after stroke compared to STD condition.

### 3.2 Resting state functional connectivity

Prior functional neuroimaging studies have revealed that behavioral recovery after stroke is associated with patterns of RS-FC within and across resting-state networks. We examined if the recovery-promoting effects of EE on sensorimotor and tactile-proprioceptive function is related to RS-FC in regions supporting the behavior in sham and stroke groups. For these comparisons, sham STD served as the control group in order to investigate if the recovery-enhancing effect of multisensory stimulation is associated with a change in functional network connectivity in relation to normal conditions.

#### 3.2.1 Enriched environment improves interregional RS-FC after stroke

**Fig. 2:**
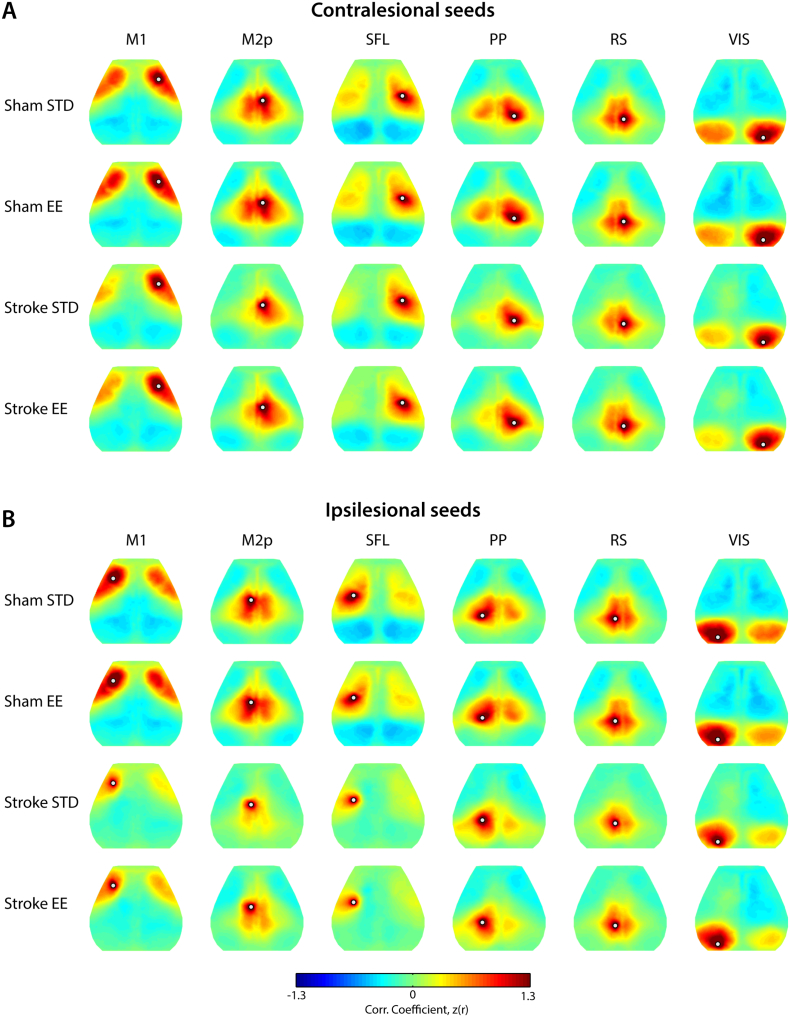
Cortical patterns of resting state functional connectivity are affected by housing condition after stroke RS-FC maps are reported between a respective seed location (white dot) and all other brain pixels for regions of interest seeds placed in (A) contralesional and (B) ipsilesional hemispheres. In sham mice (top 2 rows), regional RS-FC is not altered by housing condition. However, following stroke, housing mice in EE appears to improve inter- and intra-hemispheric RS-FC in M1 and M2p (A, B, fourth row) compared to stroke STD mice. Correlation maps (Pearson's r) of individual mice were transformed to Fisher-*z* scores prior to group averaging: sham STD, sham EE, stroke STD and stroke EE. Abbreviations: M1: primary motor, M2p: posterior secondary motor, SFL: somatosensory forelimb, PP: posterior parietal, RS: retrosplenial, VIS: visual, STD: standard environment, EE: enriched environment.

We examined RS-FC patterns in 6 predetermined seed locations for each mouse for the contralesional (Fig. 2A) and ipsilesional (Fig. 2B) hemispheres. Sham STD and sham EE mice had similar patterns of RS-FC (Fig. 2A–B, first row) with positive correlations between functionally-related cortical areas and homologous regions across hemispheres.

Following stroke, disruption in RS-FC patterns in lesioned and perilesional tissue was observed for primary motor, posterior secondary motor and somatosensory forelimb seeds placed in either hemisphere (Fig. 2A–B, third row). Seeds placed in regions remote from the lesion, e.g. in posterior parietal, and visual cortices also exhibited impaired RS-FC in the injured hemisphere after stroke. RS-FC maps in all seeds exhibited less transcallosal connectivity after stroke, i.e., loss of connectivity towards the contralateral hemisphere from the respective seeds. Housing mice in EE (Fig. 2A–B, fourth row) after stroke appears to result in increased RS-FC between perilesional regions, an effect most evident in the ipsilesional motor seed maps (Fig. 2B, M1 and M2p). In addition, mice in EE exhibit restored interhemispheric RS-FC in anterior and posterior motor regions. These observations are further quantified in the following sections.

**Fig. 3:**
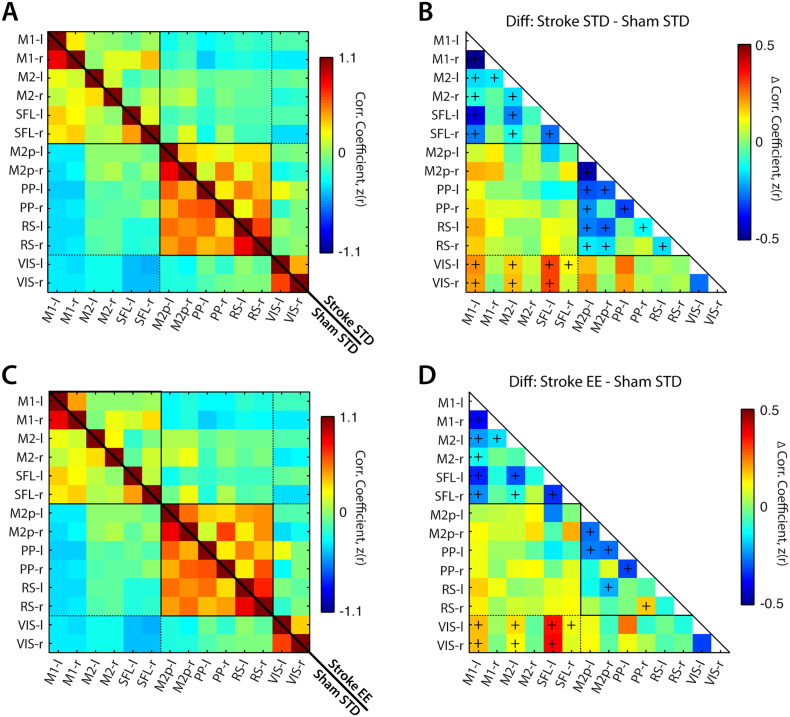
Stroke disrupts interregional RS-FC in both EE and STD groups. Quantified interregional RS-FC matrices of seeds shown in Fig. 2 for (A) sham STD (lower half) and stroke STD (upper half) and (C) sham STD (lower half) and stroke EE (upper half). Group differences in regional RS-FC are reported for (B) stroke STD minus sham STD and (D) stroke EE minus sham STD. Difference matrices were calculated by subtracting the upper triangle from the lower triangle. Abbreviations: M1: primary motor, M2 secondary motor, M2p: posterior M2, SFL: somatosensory forelimb, PP: posterior parietal, RS: retrosplenial, VIS: visual, -l: left (lesional) hemisphere, -r: right (contralesional) hemisphere, STD: standard environment, EE: enriched environment. The (+) marker indicates *p* < 0.05 following FDR correction.

**Fig. 4:**
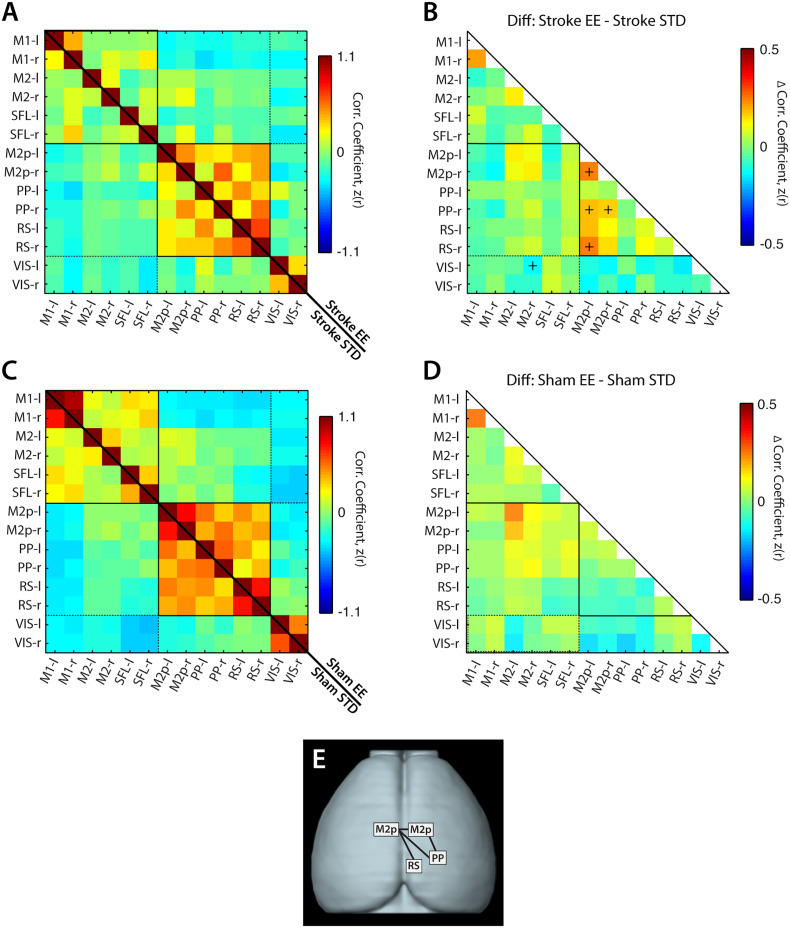
Exposure to EE leads to higher regional RS-FC strength compared to STD housing after stroke. Seed-based interregional RS-FC matrices for (A) stroke STD (lower half) and stroke EE (upper half) and (C) sham STD (lower half) and sham EE (upper half). Group differences in regional RS-FC are reported for (B) stroke EE minus stroke STD and (D) sham EE minus sham STD. Difference matrices were calculated by subtracting the upper triangle from the lower triangle. There was no effect of EE on interregional RS-FC in sham mice after stroke. (E) Schematic of brain regions exhibiting significantly enhanced RS-FC after stroke following exposure to EE. Abbreviations: M1: primary motor, M2 secondary motor, M2p: posterior M2, SFL: somatosensory forelimb, PP: posterior parietal, RS: retrosplenial, VIS: visual, -l: left (lesional) hemisphere, -r: right (contralesional) hemisphere, STD: standard environment, EE: enriched environment. The (+) marker indicates *p* < 0.05 following FDR correction.

To assess RS-FC changes in specific functional networks after stroke, RS-FC maps for each seed were compressed into matrix form (Fig. 3, Fig. 4). Compared to controls (Fig. 3), stroke followed by STD housing reduced inter- and intra-hemispheric RS-FC in most motor, somatosensory, parietal, and retrosplenial regions. Increased RS-FC was observed between visual and some motor or somatosensory cortices, reflecting a loss of anticorrelation between these regions compared to controls (Fig. 3A, B). While many of the same networks exhibited functional disruption in stroke EE mice (Fig. 3C, D), the RS-FC differences between stroke EE and sham STD were more modest than those observed in stroke STD and sham STD. For example RS-FC between left and right retrosplenial regions was significantly reduced in stroke STD (Δz(r) = − 0.17, *p* = 0.029) but not stroke EE (Δz(r) = − 0.10, *p* = 0.13) compared to sham STD mice (Figs. 3B, D). Reduced disruption of regional RS-FC was also found in the left posterior secondary motor and retrosplenial network (stroke STD: (Δz(r) = − 0.26, *p* < 0.001); stroke EE: (Δz(r) = − 0.12, *p* = 0.05)) and right posterior secondary motor and retrosplenial network (stroke STD: (Δz(r) = − 0.17, *p* = 0.005); stroke EE (Δz(r) = − 0.05, *p* = 0.34)).

The above results suggest that EE housing influenced the impact of ischemic injury on functional network connectivity. To examine which networks are most affected by housing condition after stroke, we compared RS-FC in stroke EE mice to stroke STD mice (Fig. 4A, B). Compared to STD housing conditions, EE significantly increased RS-FC of left posterior secondary motor with right posterior secondary motor (Δz(r) = 0.19, *p* < 0.001), right posterior parietal (Δz(r) = 0.15, *p* = 0.003) and right retrosplenial (Δz(r) = 0.19, *p* < 0.001), and increased RS-FC between right posterior secondary motor and right posterior parietal (Δz(r) = 0.13, *p* = 0.006) (Fig. 4A, B). Interestingly, EE also appeared to preserve the magnitude of anticorrelations between right secondary motor and left visual (Δz(r) = − 0.11, *p* < 0.001) (Fig. 4B). Because multisensory stimulation could affect RS-FC in naive animals, we also examined group difference in RS-FC in sham animals exposed to EE or STD housing. There were no significant differences in interregional RS-FC between sham STD and sham EE groups in the regions examined (Fig. 4C, D). Taken together, exposure to EE after stroke reduces the impact of ischemic injury on RS-FC disruption towards more “normal conditions” (i.e. to RS-FC values in sham STD mice). This effect is most evident between the posterior secondary motor region of the lesioned hemisphere and the contralesional posterior secondary motor, posterior parietal and retrosplenial regions of the contralesional hemisphere (Fig. 4E, Table 1).

**Table 1:** Summary of seed based interregional RS-FC. Summary of regions with significant difference in seed based interregional RS-FC. Abbreviations: M2p: posterior secondary motor, PP: posterior parietal, RS: retrosplenial, -l: left (ipsilesional), -r right (contralesional).

Seed pair	Stroke STD	Stroke EE	*p* value
M2p-l ↔ M2p-r	0.37 ± 0.03	0.56 ± 0.04	0.0007
M2p-l ↔ PP-r	0.18 ± 0.03	0.32 ± 0.03	0.0031
M2p-l ↔ RS-r	0.32 ± 0.04	0.52 ± 0.03	0.0009
PP-r ↔ M2p-r	0.43 ± 0.03	0.67 ± 0.03	0.0059

#### 3.2.2 Enriched housing influences homotopic RS-FC after stroke

**Fig. 5:**
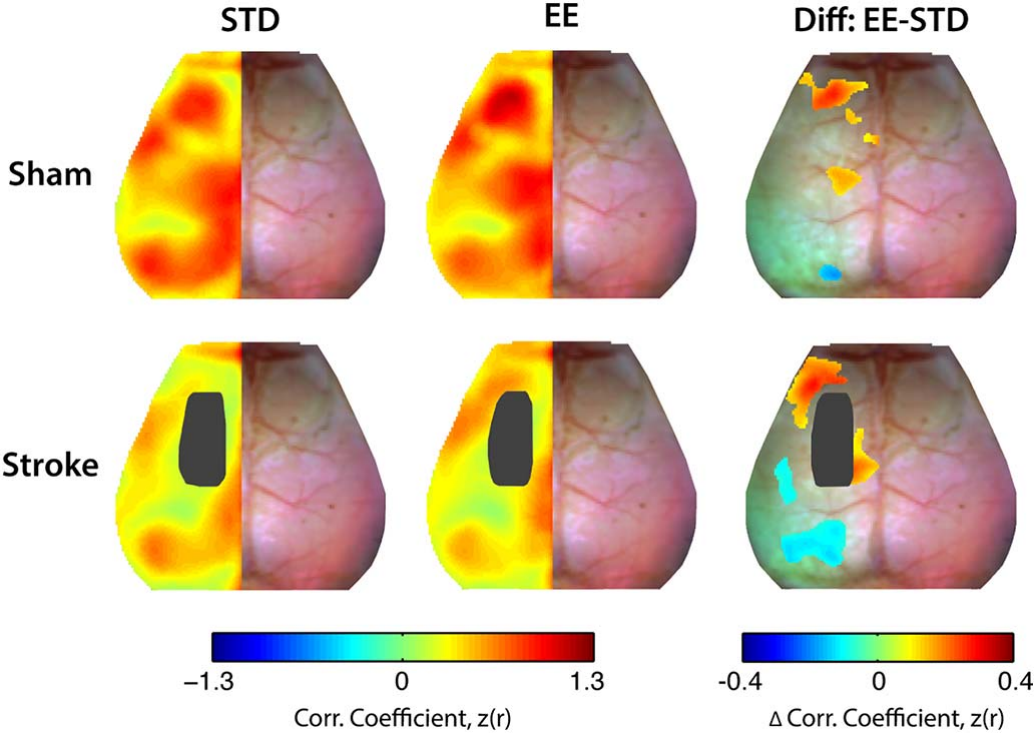
Enriched housing influences homotopic RS-FC after stroke. Group-averaged maps of homotopic RS-FC for sham (top row) and stroke (bottom row) groups after exposure to STD or EE housing. Lesion mask is indicated in black in the left hemisphere. Difference maps show changes in homotopic RS-FC between EE and STD (EE minus STD) in both the sham and stroke groups. Sham mice housed in EE exhibit enhanced homotopic RS-FC in small regions of anterior primary and secondary motor and posterior motor and somatosensory hindlimb cortices. Mice with stroke housed in EE showed increased homotopic RS-FC over larger regions of anterior primary and secondary motor cortex, posterior secondary motor cortex, and reduced homotopic RS-FC between parts of barrel somatosensory cortices and larger regions of visual cortex. STD: standard environment, EE: enriched environment, Diff: difference. Maps of homotopic RS-FC differences were thresholded at *p* < 0.05, uncorrected.

Disruptions in homotopic RS-FC are a strong proxy for behavioral deficits after clinical and experimental stroke (Bauer et al., 2014, Carter et al., 2010, Silasi and Murphy, 2014, van Meer et al., 2010). To examine how our induced stroke model affected cortical homotopic RS-FC in each mouse, spontaneous activity in every pixel within our field-of-view was correlated with its contralateral homolog to produce a map of homotopic RS-FC. Group-level maps are visualized in the left (ipsilesional) hemisphere (Fig. 5). In the control group (sham STD), high positive homotopic correlations were observed in primary motor (z(r) = 0.65 ± 0.04), posterior parietal (z(r) = 0.69 ± 0.02), retrosplenial (z(r) = 0.87 ± 0.03) and visual (z(r) = 0.68 ± 0.03) areas, while more modest correlations in RS-FC were seen between homotopic somatosensory areas (forelimb (z(r) = 0.48 ± 0.04) and barrel (z(r) = 0.45 ± 0.12)) (Fig. 5 top row). Sham mice housed in EE exhibit enhanced homotopic RS-FC compared to sham STD mice in parts of frontal association (Δz(r) = 0.17, *p* = 0.002), secondary motor (Δz(r) = 0.19, *p* = 0.002) and posterior primary motor (Δz(r) = 0.14, *p* = 0.004) (Fig. 5 top row).

**Supplementary Fig. 4:**
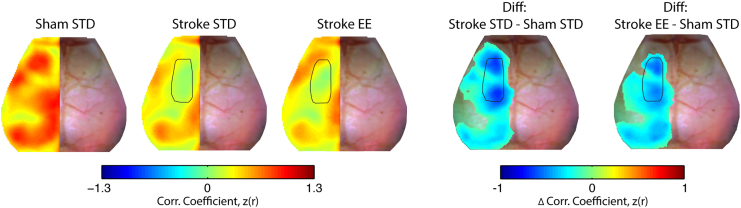
Effect of stroke on homotopic RS-FC. Group-averaged maps of homotopic RS-FC for sham STD and stroke groups after exposure to STD or EE housing. While focal ischemia results in a dramatic reduction of homotopic RS-FC in both groups, functional disruption is reduced following EE exposure. Difference maps were thresholded at *p* < 0.05, uncorrected.

Following stroke, significantly reduced homotopic RS-FC ranging from 20 to 70% across our field-of-view was observed in the both groups (Fig. 5 bottom row), particularly within the lesioned and perilesional motor areas (Fig. S4; Table S1). However, appreciable differences in homotopic RS-FC strength are observed in motor regions between stroke STD and stroke EE groups (Fig. 5, bottom row). Mice housed in EE after stroke showed increased homotopic RS-FC compared to STD mice in frontal association (Δz(r) = 0.21, *p* = 0.009), perilesional primary motor (Δz(r) = 0.19, *p* = 0.015) and notable in the posterior secondary motor cortex (Δz(r) = 0.15, *p* < 0.001), while a decrease in homotopic RS-FC was found within barrel somatosensory (Δz(r) = − 0.10, *p* = 0.013) and visual cortex (Δz(r) = − 0.11, *p* = 0.001).

Regional homotopic RS-FC strength in naïve mice was also enhanced by multisensory stimulation. Interestingly, some of these regions, for example anterior and lateral portions of primary and secondary motor cortices, overlap with those observed in the stroke mice. In addition, sham mice housed in EE present enhanced homotopic RS-FC in in somatosensory hindlimb and posterior primary motor regions, both regions located in the lesion territory of the stroke groups (Fig. 5).

#### 3.2.3 Multisensory stimulation increases regional node degree after focal ischemia

**Fig. 6:**
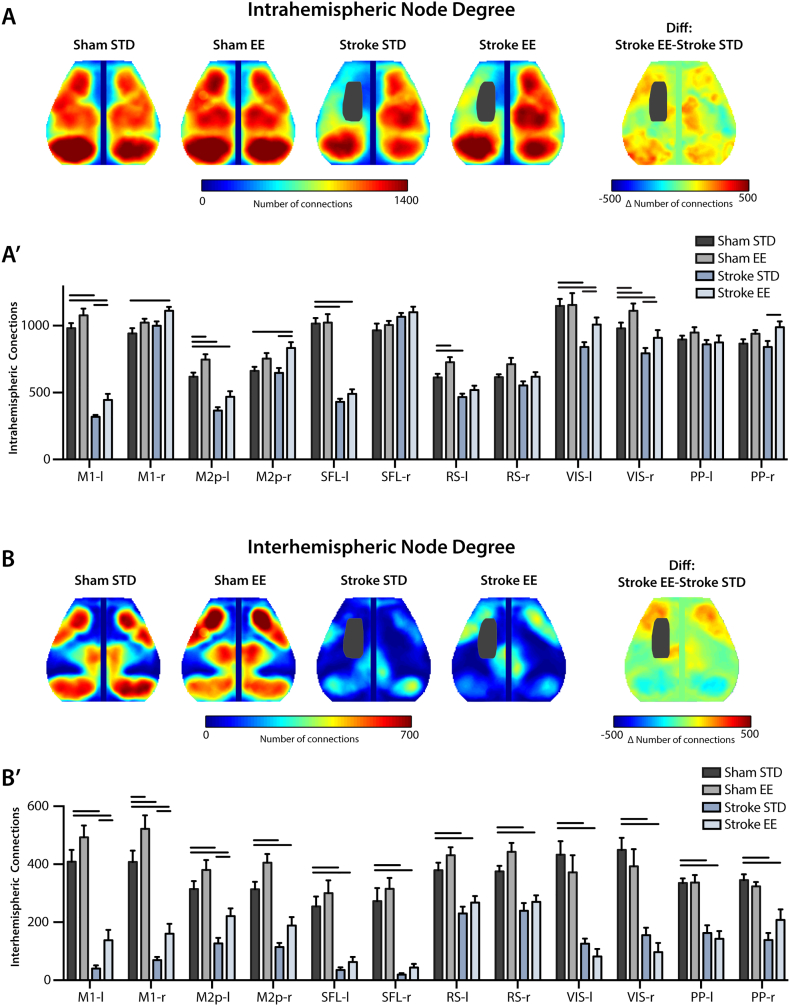
Exposure to EE leads to increased node degree compared to STD housing after stroke Group-averaged maps of node degree in sham and stroke groups after housing in STD or EE. The number of functional connections were determined for each pixel by thresholding that pixel's RS-FC map at z(r) = 0.4, and counting all pixels above that threshold in the ipsilateral (A), or contralateral (B) hemisphere to that pixel. (A′, B′) Quantification of node degree in regions defined by atlas assignments defined in Fig. 1. Compared to sham STD, stroke significantly diminished intrahemispheric node degree in the lesioned hemisphere, and reduced interhemispheric node degree in all regions in both hemispheres. Compared to STD housing, exposure to EE after stroke significantly increased intrahemispheric node degree in ipsilesional motor and visual regions, and interhemispheric posterior secondary motor, parietal, and visual regions. Housing in EE also resulted in increased post-stroke interhemispheric node degree in ipsilesional and contralesional primary motor, and ipsilesional posterior secondary motor cortex. Abbreviations: M1: primary motor, M2p: posterior secondary motor, SFL: somatosensory forelimb, PP: posterior parietal, RS: retrosplenial, VIS: visual, STD: standard environment, EE: enriched environment. Diff: difference. Horizontal bars indicate significant differences (*p* < 0.05, following FDR correction).

In addition to examining pairwise differences in regional RS-FC, we also examined the number of functional connections (node degree) that a given region had with other ipsilateral (intrahemispheric) (Fig. 6A) or contralateral (interhemispheric) brain pixels (Fig. 6B). In the control group (sham STD) high intrahemispheric node degree was observed for most brain regions, particularly in motor (primary motor: 980 ± 38; posterior secondary motor: 620 ± 31), somatosensory (1016 ± 41), posterior parietal (897 ± 29) and visual (1148 ± 51) regions (Sham STD, Fig. 6A, A′). More heterogeneity in connectivity strength was observed in the interhemispheric node degree maps. For example, some brain regions exhibiting high intrahemispheric node degree also exhibited strong interhemispheric node degree (primary motor: 409 ± 41, posterior parietal: 336 ± 16, visual: 433 ± 46). But others, for example somatosensory regions, showed relatively little interhemispheric node degree (255 ± 34; Fig. 6B, B′). Node degree for sham STD and sham EE groups were generally comparable. However, mice in EE had increased intrahemispheric node degree in left posterior secondary motor (ΔND*i* = 127, *p* = 0.041) and right visual (ΔND*i* = 133, *p* = 0.032; Fig. 6A′) and increased interhemispheric node degree in right primary motor (ΔND*c* = 114, *p* = 0.012; Fig. 6B′).

When comparing sham STD to stroke STD, stroke significantly diminished intrahemispheric node degree by 41–61% in perilesional motor and somatosensory regions (*p* < 0.001) (Fig. 6A′). In the ipsilesional hemisphere, remote areas like visual (26%, *p* < 0.001) and retrosplenial (24%, *p* = 0.011) also showed appreciable reduction (Fig. 6A′). However, intrahemispheric node degree was not appreciably reduced in the posterior parietal region (*p* = 0.53) of the left hemisphere (Fig. 6A′). In the contralesional hemisphere only visual cortices had reduced intrahemispheric node degree after stroke (stroke STD, 19%, *p* < 0.001; Fig. 6A′). As indicated by the homotopic RS-FC maps, stroke significantly reduced interhemispheric node degree in all regions in both hemispheres (20–90%, *p* < 0.001) — suggesting a general reduction of temporal coherence between hemispheres across most regions of the cortex (stroke STD, Fig. 6B, B′).

Although a general reduction in intrahemispheric and interhemispheric node degree was observed in the stroke EE group compared to controls, when comparing stroke EE to stroke STD, significant increases in intrahemispheric node degree were found in several brain regions. For example, left primary motor (ΔND*i* = 127, *p* = 0.017), left visual (ΔND*i* = 166, *p* = 0.002), and right visual (ΔND*i* = 116, *p* = 0.03) regions (Fig. 6A′) all exhibited a higher number of intrahemispheric functional connections compared to stroke STD mice. Further, in right posterior secondary motor (ΔND*i* = 187, *p* < 0.001), posterior parietal (ΔND*i* = 148, *p* = 0.006) intrahemispheric node degree was also increased above stroke STD. Increased interhemispheric node degree was observed in the stroke EE group in both left primary motor (ΔND*c* = 98, *p* = 0.012) and right primary motor (ΔND*c* = 91, *p* = 0.019), as well as in left posterior secondary motor (ΔND*c* = 94, *p* = 0.015) (Fig. 6B, B′), the same networks exhibiting increased RS-FC in the regional analyses of Fig. 2, Fig. 3, Fig. 4, Fig. 5. Taken together these results show that housing mice in EE after stroke improves interhemispheric RS-FC of perilesional motor areas, improves intrahemispheric RS-FC within perilesional primary motor, and enhances RS-FC in the posterior secondary motor and posterior parietal of the intact hemisphere.

### 3.3 Changes in PV-immunoreactive cells

**Fig. 7:**
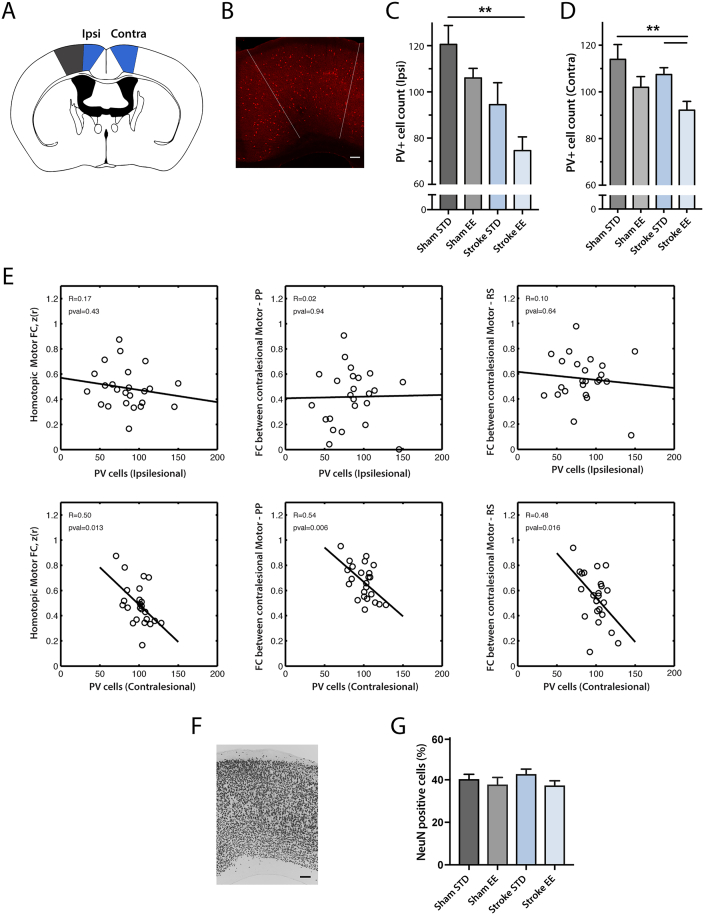
The number of parvalbumin (PV) immunoreactive neurons is reduced in regions exhibiting enhanced RS-FC following EE exposure. (A) Area of interest (blue) and lesioned area (grey) in a horizontal section of the mouse brain. (B) Representative micrograph showing PV-immunoreactive cells in the contralateral posterior motor cortex (scalebar = 100 μm). Recovery of RS-FC in mice housed in EE is associated with a decrease in PV-immunoreactive cells in (C) ipsilesional and (D) contralesional motor cortex. (E) RS-FC vs parvalbumin neuron number. In the contralesional hemisphere, the number of parvalbumin immunoreactive neurons correlated with RS-FC between motor and posterior parietal regions, and between motor and retrosplenial cortex after stroke. (F) Representative micrograph showing NeuN positive cells of the posterior motor cortex (scalebar = 100 μm). (G) There is no difference in NeuN positive cells between the stroke groups in the contralesional posterior motor cortex as determined by area covered by cells in percent (*p* = 0.47, FDR corrected). Abbreviations: M2p: posterior secondary motor, PP: posterior parietal, RS: retrosplenial. STD: standard environment, EE: enriched environment (unit: cells/mm^2^). Horizontal bars indicate significant differences (**p < 0.01 following FDR correction).

**Supplementary Fig. 5:**
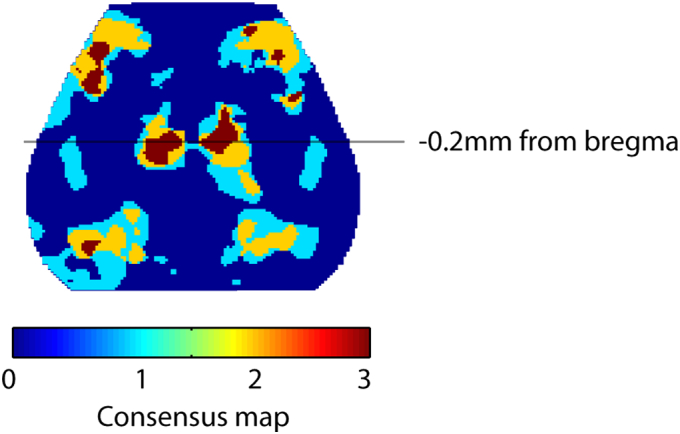
Consensus difference map for Stroke EE vs Stoke STD connectivity measures. Difference maps for homotopic FC, interhemispheric- and intrahemispheric node degree were thresholded at an uncorrected *p*-value of 0.05 and overlaid on the same image. Connectivity differences in posterior motor regions were shared across all 3 methods in both hemispheres. The line at − 0.2 mm from bregma marks the approximate location for histological quantification of PV cell count in Fig. 7.

Earlier studies indicated that PV immunoreactivity is associated with functional recovery after stroke (Alia et al., 2016, Zeiler et al., 2013). The effect of EE on stroke recovery was most pronounced in posterior motor cortex as demonstrated by consensus differences in RS-FC across three different analysis methods (homotopic-, interhemispheric- and intra-hemispheric node degree, Fig. S5). Decreases in PV-immunoreactivity have been associated with improved behavioral recovery (Alia et al., 2016, Zeiler et al., 2013). Given the improved behavioral performance in the EE group, we examined if the observed changes in motor cortical reorganization related to the number of PV immunoreactive cells in motor regions exhibiting increased RS-FC across all 3 methods (Fig. 7A–B). Results among groups were analyzed independently for the left and right hemispheres.

Housing in EE after stroke led to reduced levels of PV^+^ cells in bilateral posterior motor cortices ranging from 20 to 38% compared to controls (sham STD). In the ipsilesional hemisphere (Fig. 7C), reduced levels of PV^+^ cells were seen in stroke EE mice (75 ± 6) compared to sham STD (121 ± 8, *p* = 0.002). In the contralesional hemisphere (Fig. 7D) there was a significant decrease in PV^+^ cells between sham STD (114 ± 6) and stroke EE (92 ± 4, *p* = 0.003) and between stroke STD (108 ± 3) and stroke EE (92 ± 4, *p* = 0.003).

Fig. 7E shows RS-FC of posterior motor cortex and other brain regions in relation to numbers of PV-immunoreactive cells in those regions following stroke. PV cell counts in contralesional posterior motor correlated with homotopic RS-FC between posterior motor regions (*R* = 0.50, *p* *=* 0.013), and with interregional RS-FC in the contralesional hemisphere between posterior motor and posterior parietal (*R* = 0.54, *p* = 0.006) and between posterior motor and retrosplenial (*R* = 0.48, *p* = 0.016). In contrast, PV counts in the ipsilesional hemisphere did not correlate with interregional RS-FC between homotopic posterior motor (*R* = 0.17, *p* *=* 0.43), contralesional posterior motor and posterior parietal (*R* = 0.02, *p* = 0.94) or contralesional posterior motor and retrosplenial (*R* = 0.1, *p* = 0.64) cortices.

NeuN positive neurons were quantified to assess whether the decrease in PV immunoreactivity in the contralesional posterior motor cortex was due to a general loss of neurons. There was no significant difference in the area covered by NeuN^+^ cells between any of the experimental groups (Sham STD (40 ± 2%), Sham EE (38 ± 3%), stroke STD (43 ± 2%) and stroke EE (38 ± 2%)).

## 4 Discussion

This study shows for the first time that recovery of lost neurologic function after housing mice in an EE is associated with enhanced regional RS-FC in experimental stroke. The effect of post-stroke housing in an EE on RS-FC is most prominent in primary and secondary motor cortices, and in posterior-parietal, retrosplenial, and visual cortices. Furthermore, this study reveals that changes in RS-FC of these regions are correlated with a decrease in PV-immunoreactivity. We discuss these findings below and propose that multisensory stimulation provided by EE after stroke remodels the intrinsic functional network architecture of the brain in a manner that enhances recovery of sensorimotor function (specifically tactile-proprioception), and that PV-immunoreactive cells are important modulators of this plasticity process. Finally, we will discuss the implication of this study for clinical rehabilitation after stroke.

### 4.1 Effects of stroke on RS-FC

Compared to controls, mice subjected to stroke, regardless of housing in STD or EE environments, exhibited a dramatic reduction in RS-FC in motor and somatosensory cortex, i.e. regions directly affected by photothrombosis. We also observed regions of diaschisis, indicating a disconnection of networks (Von Monakow, 1914). Specifically, focal ischemia in left motor and somatosensory regions indirectly affected remote cortical areas such as ipsi- and contra-lesional retrosplenial, posterior parietal and visual cortices (Fig. 3, Fig. 6 and S3). These local and distant effects on RS-FC have been observed using optical (Bauer et al., 2014) and fMRI (van Meer et al., 2012, van Meer et al., 2010) techniques in rodent models in the acute phase after stroke, and correlate with functional impairment. Further, acute deficits in regional inter- and intra-hemispheric RS-FC can serve as proxies for behavior, which has particular relevance to linking with stroke studies in stroke patients (Carter et al., 2010, Grefkes and Ward, 2014, Rehme and Grefkes, 2013, Siegel et al., 2016).

RS-FC between mirrored brain pixels informs the amount of interhemispheric disconnection between nominally homotopic regions following stroke, an important measure of acute functional disruption in human subjects. However, contralateral homologues of pixels might not be necessarily homotopic. For example, small differences in interhemispheric anatomy will disrupt the midline symmetry assumed in our homotopic RS-FC measures. Further, cortical remodeling following focal damage can appreciably alter the relative spatial locations of homologous brain regions. While we did not examine cortical remapping directly (e.g. through peripheral stimulation of infarcted brain regions) the measures of node degree were performed over our entire field of view, and are thus sensitive to the formation of new, but displaced, homotopic functional connections (provided those connections are above the threshold). Together, the measures of RS-FC employed in the study account for acute functional disruption of homotopic and other regions, and the possibility of functional remodeling over the 2 week recovery period.

### 4.2 Effects of enriched environment on functional connectivity after stroke

In naïve rodents, exposure to EE can increase cortical responsiveness through cognitive, sensory and motor stimulation. EE has an effect on multiple molecular endpoints, including an increase in synapse number and neurogenesis (Nithianantharajah and Hannan, 2006). These changes can be reflected in the subtle to moderate improvements of homotopic and node degree RS-FC in motor cortices that we observe in sham EE mice compared to sham STD. We speculate that EE induced changes in the healthy brain may occur over a different time scale compared to mice with stroke, but likely involves many of the same regions that exhibit enhanced RS-FC after stroke (e.g. regions involved in sensorimotor function and control of movements).

After stroke, multisensory stimulation enhances recovery of RS-FC at the level of large-scale neuronal networks. For example, stroke EE mice show significantly higher homotopic RS-FC strength in perilesional motor areas (primary motor and posterior secondary motor; Fig. 5). Likewise, perilesional motor regions exhibited a larger number of intrahemispheric functional connections in stroke EE mice than mice housed in STD cages (Fig. 6A), findings that align well with previous reports showing dramatic remodeling of motor maps occurring within ipsilesional brain regions of rats (Starkey et al., 2012).

Our observation of enhanced interhemispheric network communication following multisensory stimulation suggests that EE facilitates a return to normal function through re-establishment of RS-FC within and across many networks (Fig. 5, Fig. 6B). While the present findings provide further evidence that homotopic RS-FC of injured brain regions is associated with better outcome, increased RS-FC was not just observed in ipsi- and contra-lesional motor regions. For example, the contralesional parietal region exhibited increased intra- and inter-hemispheric functional connectivity, in seed-based RS-FC analysis (Fig. 4) and analysis of node degree, in stroke EE mice compared to stroke STD mice (Fig. 6).

Increased functional connectivity in contralesional motor regions could in part be due to wide-spread disinhibition (Dijkhuizen et al., 2003, Mohajerani et al., 2011). As suggested in (van Meer et al., 2012, van Meer et al., 2010), contralesional remodeling can potentially reflect functional reorganization through active compensation with the intact forelimb during sensorimotor recovery of the affected forelimb. Restoration of new functional connections requires an anatomical substrate to facilitate synaptic communication. For example, after large strokes, contralesional contribution to recovery involves sprouting of axons from contralesional cortico-spinal tract to the denervated side at the level of the spinal cord after Nogo-A treatment (Lindau et al., 2014, Wahl et al., 2014). Enhanced axonal outgrowth and spine density in perilesional regions (Carmichael et al., 2017) could also provide new structural connectivity for supporting increased RS-FC observed here.

We found that mice housed in STD cages after stroke exhibit decreased homotopic RS-FC and interhemispheric node degree in visual cortices (Fig. S3, Fig. 6B). After exposure to EEs this effect appears to be increased (Fig. 5, Fig. 6B). Interestingly, intrahemispheric node degree analysis also indicated a shift towards increased intrahemispheric connectivity of visual regions in both hemispheres (Fig. 6A). This may be an effect of overall changes in sensory integration and processing, or an increased intrahemispheric contribution to functional recovery (Bannister et al., 2015, Cramer et al., 1997, Kopp et al., 1999). Primary visual cortices mainly evoke direct transcallosal excitation of neurons in the opposite visual cortex (Restani et al., 2009). The opposite effect of EE on homotopic RS-FC between motor and visual cortices can be due to fundamental differences in transcallosal homotopic excitation/inhibition between these regions. For example, net interhemispheric inhibition between the sensorimotor cortices, influences fine motor control, and somatosensory processing (Palmer et al., 2012).

### 4.3 Improvement in tactile-proprioception function is associated with RS-FC in posterior parietal and secondary motor regions

Housing in an EE had a particularly strong effect on improving tactile-proprioceptive paw function. The stroke lesion was positioned in the left sensorimotor cortex, encompassing most of primary motor, somatosensory hindlimb, and parts of secondary motor and somatosensory forelimb areas. Lesions to these brain regions reliably produce robust and persistent proprioceptive dysfunction in both contralesional limbs two weeks after stroke (De Ryck et al., 1992, Quattromani et al., 2014). This deficit is particularly prominent in the absence of visual input, and almost completely restored after housing in an EE (De Ryck et al., 1992, Madinier et al., 2014). This implies that EE enhances recovery of particular networks involved in sensory integration such as touch and proprioception. The tactile-proprioceptive limb placement test is a highly sensitive marker of recovery of somatosensory function following stroke (Dukelow et al., 2012) and clinically relevant since 50–80% of stroke patients have persistent somatosensory impairments including proprioception (Carey and Matyas, 2011, Kim and Choi-Kwon, 1996). In humans, activation of higher-order sensorimotor cortices, specifically dorsal premotor and the supramarginal gyrus (SMG) portion of the posterior parietal cortex, appears to play a key role in interpreting tactile sensory information and the perception of limb position, and has been proposed to incorporate a “command apparatus” for movement (Mountcastle et al., 1975, Rathelot et al., 2017). In addition, decreased right SMG function in stroke patients is associated with decreased proprioceptive function (Ben-Shabat et al., 2015). The posterior parietal cortex in mice and rats is considered homologous to the primate posterior parietal cortex based on anatomical studies (Broussard, 2012). As in humans, the mouse posterior parietal integrates multiple modes of sensory inputs including auditory, visual and somatosensory regions (Zingg et al., 2014). This is in line with our finding, where these cortical areas (left and right posterior secondary motor) display high RS-FC with the posterior parietal seed in control animals (Fig. 4). Also, posterior parietal does appear to be required for visual sensorimotor decision tasks in mice (Goard et al., 2016). It has been suggested that secondary motor acts as an important link between multisensory inputs and subsequent motor output (Yamawaki et al., 2016). The distinct cortico-cortical structural and functional connections between retrosplenial, posterior secondary motor and posterior parietal cortex (Figs. 2, S1) might provide a means for relaying information from the dorsal hippocampus to neocortical regions involved in the diverse aspects of sensorimotor integration and motor control (Yamawaki et al., 2016). In the cortex, secondary motor projects back to posterior parietal, sensory and retrosplenial cortices, completing a relatively complex, reciprocally-connected network between these areas for processing sensory input (Barthas and Kwan, 2016).

At 14 days after stroke, our interregional and node degree analysis revealed that, compared to STD, EE increased RS-FC among these particular networks relevant for recovery of proprioception (Ben-Shabat et al., 2015) and touch (Carey et al., 2016) in stroke patients. We therefore propose that RS-FC changes observed in the secondary motor cortices, contralesional posterior parietal and retrosplenial regions could be instrumental in the marked improvements in tactile-proprioceptive function observed after stroke in the EE group.

### 4.4 Association of PV cells with functional connectivity changes after stroke

Multisensory stimulation provided by EE modulates multiple biological mechanisms that could lead to accelerated recovery following focal injury. Among them, cortical GABA interneurons have been shown to contribute to increasing the excitatory/inhibitory ratio (Alwis and Rajan, 2013). PV/GABA neurons are of critical importance for plasticity and information processing in the cortical micro columns (Freund and Katona, 2007) and are affected by enriched housing conditions. In the rat auditory cortex, EE reduced expression of GABA-A receptor subunits and decreased GABAergic inhibition (Zhou et al., 2011). In the cat EE decreased the number of inhibitory synapses in the visual system (Beaulieu and Colonnier, 1987). Further, decreased levels of hippocampal PV expression, induced by housing mice in EE, enhanced structural synaptic plasticity and improved memory consolidation and retrieval (Donato et al., 2013).

Our findings of reduced PV immunoreactivity after stroke are in line with these data. We found a decreased number of PV-immunoreactive cells at 14 days post stroke, compared to the control animals, in both left and right posterior motor cortex (Fig. 7). EE further reduced PV immunoreactivity in the contralesional hemisphere compared to stroke STD. The decrease in PV could be due to the enhanced degradation of perineuronal nets surrounding the PV/GABA interneurons induced by EE (Balmer, 2016, Hsieh et al., 2016, Madinier et al., 2014, Quattromani et al., 2017, Yamada et al., 2015). We also found that the number of PV cells in the contralesional motor cortex correlated with RS-FC between homotopic motor cortices, and the intrahemispheric RS-FC between posterior motor, posterior parietal and retrosplenial cortices. This suggest that that the decrease in PV immunoreactivity could lead to decreased tonic inhibition by cortical PV/GABA interneurons, enhanced plasticity in the cortical micro columns, and subsequently to restoration of functional networks.

### 4.5 Clinical implications

It has been proposed that the tissue surrounding small stroke lesions is inhibited by input from the contralesional hemisphere, thereby limiting recovery (interhemispheric imbalance model) (Plow et al., 2016). Hence, decreasing contralesional hemispheric excitability, for example using TMS or tDCS, may improve functional outcome (Boddington and Reynolds, 2017). In contrast, in patients with large infarcts, contralesional brain areas such as primary motor (Lotze, 2006) and in particular the dorsal premotor cortex (Bestmann et al., 2010, Plow et al., 2016) can contribute to better functional outcome (Di Pino et al., 2014, Rehme et al., 2011). Here, facilitation of excitability of the contralesional cortex by neuromodulation may promote functional recovery (Plow et al., 2016). In relation to somatosensory impairment and recovery, better performance has been associated with task-related de-activation of contralesional thalamus, and with de-activation of ipsilesional primary somatosensory cortex (adjacent to the SI hand area activated in healthy controls) (Carey et al., 2011), further highlighting a role for rebalancing of activity across hemispheres. The infarct in our stroke model includes large parts of primary sensorimotor areas. Significant post-stroke remodeling occurs in the contralesional hemisphere within primary motor, posterior secondary motor, and posterior parietal cortices when animals are subjected to multisensory stimulation provided by EE. These results imply that in patients with large hemispheric lesions, contralesional posterior motor cortex and associated secondary and posterior parietal cortex could potentially be targeted by multimodal sensory stimulation and/or neuromodulation using tools such as TMS or tDCS during rehabilitation.

## 5 Conclusions

Our results show that multisensory stimulation provided through housing in enriched environments (EE) results in a more rapid return to normal brain function in the mouse after stroke. Housing in EE significantly improved lost tactile-proprioceptive function compared to mice housed in standard (STD) cages. The improved behavioral performance of mice housed in EEs was associated with improved homotopic RS-FC between motor cortices. Further, mice exposed to enriched housing also exhibited increased RS-FC in secondary motor, posterior parietal and retrosplenial cortices, regions involved in multisensory integration for initiation and coordination of movements. Multisensory stimulation also leads to the modulation of PV/GABA cell activity, which might contribute to a recovery-enhancing effect observed herein. Our data imply that targeting neural circuitry involving spared motor regions, specifically higher-order sensorimotor cortices involved in motor commands, by multimodal sensory stimulation and neuromodulation could support rehabilitation of stroke patients.

The following are the supplementary data related to this article.

Supplementary Table 1 Effect of stroke on regional homotopic functional connectivity.
Quantification of homotopic RS-FC for in regions defined by atlas assignments in Fig. 1. FA: frontal association, M1: primary motor, M2p: posterior secondary motor, SFL: somatosensory forelimb, BA: somatosensory barrel, PP: posterior parietal, RS: retrosplenial, VIS: visual. All *p*-values are FDR corrected. Supplementary Table 1
