# Supplementary material for: Multisensory stimulation improves functional recovery and resting-state functional connectivity in the mouse brain after stroke
Source: Neuroimage Clin. 2017 Dec 2;17:717–30. doi: 10.1016/j.nicl.2017.11.022 (PMC5726755; doi:10.1016/j.nicl.2017.11.022)
Supplement: Supplementary Table 1 — Effect of stroke on regional homotopic functional connectivity. Quantification of homotopic RS-FC for in regions defined by atlas assignments in Fig. 1. FA: frontal association, M1: primary motor, M2p: posterior secondary motor, SFL: somatosensory forelimb, BA: somatosensory barrel, PP: posterior parietal, RS: retrosplenial, VIS: visual. All p-values are FDR corrected. [file mmc1.docx]

| **Region** | **Sham STD (A)** | **Stroke STD (B)** | **Stroke EE (C)** | ***p* value (A vs B)** | ***p* value (A vs C)** |
| --- | --- | --- | --- | --- | --- |
| **FA** | 0.43±0,03 | 0.30±0.03 | 0.43±0.05 | 8.8×10^-3^ | 0.96 |
| **M1** | 0.65±0.04 | 0.19±0.09 | 0.27±0.05 | 1.9×10^-10^ | 2.5×10^-5^ |
| **M2p** | 0.50±0.03 | 0.25±0.03 | 0.37±0.05 | 1.2×10^-5^ | 0.04 |
| **SFL** | 0.48±0.04 | 0.20±0.02 | 0.19±0.02 | 2.7×10^-7^ | 7.7×10^-8^ |
| **BA** | 0.45±0.03 | 0.30±0.3 | 0.23±0.02 | 6.9×10^-4^ | 5.6×10^-6^ |
| **PP** | 0.69±0.02 | 0.41±0.04 | 0.39±0.05 | 1.4×15^-8^ | 5.1×10^-5^ |
| **RS** | 0.87±0.03 | 0.70±0.03 | 0.73±0.02 | 8.1×10^-4^ | 8.8×10^-4^ |
| **VIS** | 0.68±0.03 | 0.46±0.03 | 0.36±0.03 | 3.2×10^-5^ | 8.3×10^-8^ |

**Supplementary Table 1. Effect of stroke on regional homotopic functional connectivity.**

Quantification of homotopic RS-FC for in regions defined by atlas assignments in Fig. 1. FA: frontal association, M1: primary motor, M2p: posterior secondary motor, SFL: somatosensory forelimb, BA: somatosensory barrel, PP: posterior parietal, RS: retrosplenial, VIS: visual. All p-values are FDR corrected.
